# A dinosaur community composition dataset for the Late Cretaceous Nemegt Basin of Mongolia

**DOI:** 10.1016/j.dib.2017.11.086

**Published:** 2017-12-06

**Authors:** G.F. Funston, S.E. Mendonca, P.J. Currie, R. Barsbold

**Affiliations:** a Department of Biological Sciences, CW 405 Biological Sciences Building, University of Alberta, Edmonton, AB, Canada T6G 2E9; b Department of Earth and Atmospheric Sciences, University of Alberta, 1-26 Earth Sciences Building, Edmonton, AB, Canada T6G 2E3; c Institute of Paleontology and Geology, Mongolian Academy of Sciences, Box-46/650, Ulaanbaatar 15160, Mongolia

## Abstract

Dinosaur community composition data for eleven fossil localities in the Late Cretaceous Nemegt Basin of Mongolia are compiled from field observations and records in the literature. Counts were generated from skeletons and represent numbers of individuals preserved in each locality. These data were used in the analyses of Funston et al. [1] “Oviraptorosaur anatomy, diversity, and ecology in the Nemegt Basin” in the Nemegt Ecosystems Special Issue of Palaeogeography, Palaeoclimatology, Palaeoecology, where the results are discussed.

**Specifications Table**

**Table:** 

Subject area	*Evolutionary Biology*
More specific subject area	*Palaeontology and Palaeoecology*
Type of data	*Tables, Interactive map*
How data was acquired	*Field observations and literature survey*
Data format	*Raw tables and .kmz files for Google Earth*
Experimental factors	*None*
Experimental features	*None*
Data source location	*Nemegt Basin, Western Gobi Desert, Mongolia*
Data accessibility	*Within this article and as*Supplementary material

**Value of the data**

• We combined new field observations with an extensive literature survey, compiling an unparalleled community composition dataset for dinosaur palaeontology.
• The dataset includes nearly 500 skeletons identifiable to species, which allows for detailed comparison of community composition with other ecosystems around the globe.
• Some of the data were collected using GPS, and the map generated from this data allows for examination of finer-scale spatial relations of the skeletons possibly related to taphonomy or palaeoecology.

## 1 Data

**Fig. 1:**
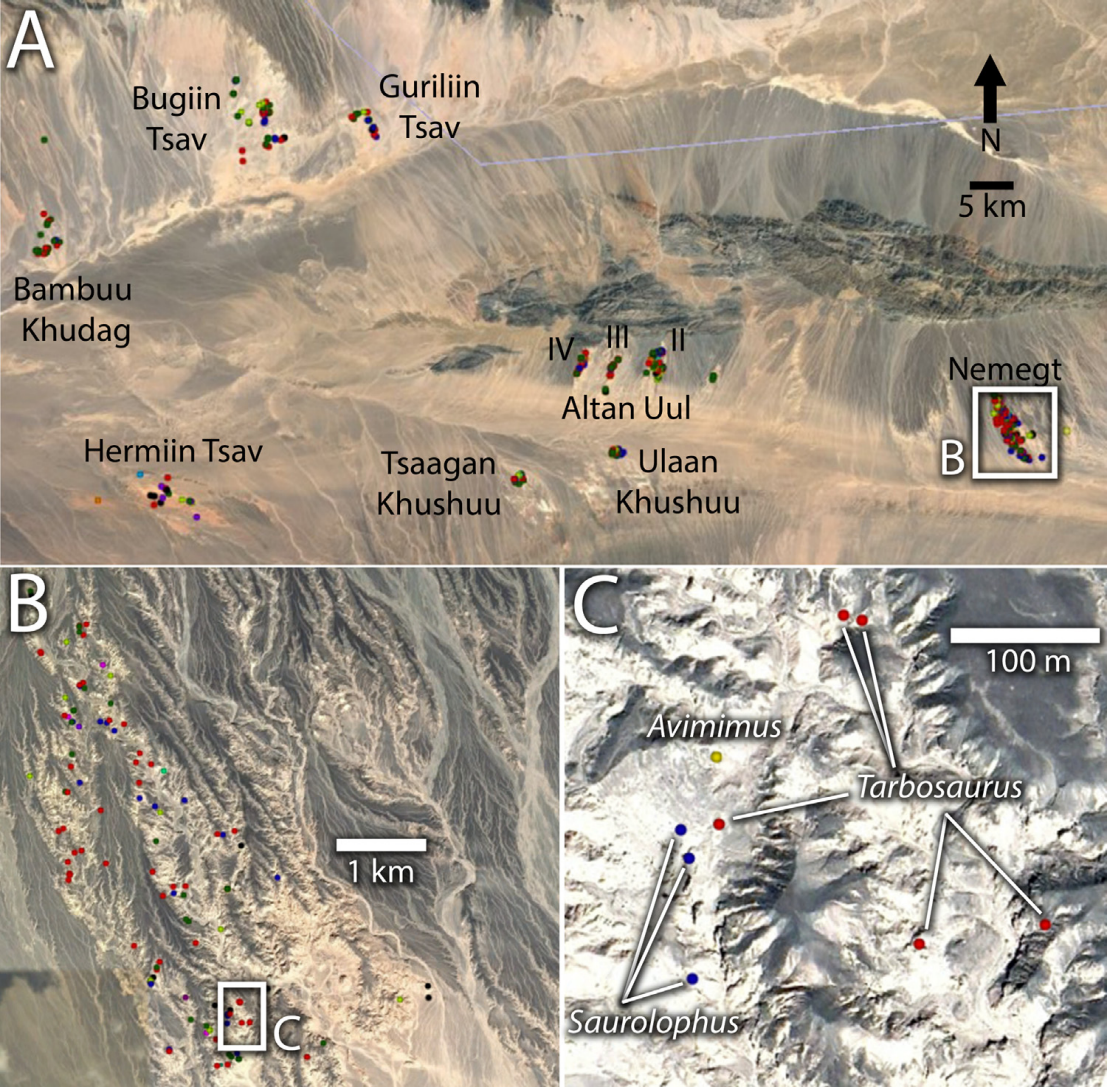
Example images from interactive map data. Overview (A) of Nemegt Basin, showing localities and clusters of GPS-acquired datapoints. Detail of Nemegt Locality (B) showing distribution of skeletons along sayrs. Close-up (C) of Central Sayr, showing fine-scale spatial resolution of data, and colour coding of different taxa. Scales as indicated, North is up in all images.

The data tables presented (Table 1 and Supplementary Table 1) are whole-number counts of the number of skeletons of each type of dinosaur at each locality. There are two tables: the first records occurrence of specimens identifiable to the species-level, the second groups specimens by superfamily. The second table therefore includes some specimens that are not identifiable to species, but are still informative for overall community composition. The columns represent a taxon, and the rows represent localities. The interactive map (Fig. 1; Supplementary File 1) is a Google Earth (.kmz) file, which has GPS locations of 358 dinosaur skeletons, colour coded by taxon.

**Table 1:** Superfamily-level dinosaur community composition data for 11 Mongolian localities.

**Community**	**Oviraptorosaurs**	**Ankylosaurs**	**Tyrannosaurs**	**Hadrosaurs**	**Ornithomimids**	**Therizinosaurs**	**Pachycephalosaurs**	**Deinonychosaurs**	**Ceratopsians**	**Alvarezsaurs**	**Sauropods**
Hermiin Tsav	21	14	14	1	2	0	0	1	36	3	2
Khulsan	4	2	0	1	0	0	1	1	3	0	0
Bambuu Khudag	0	1	8	1	15	0	0	0	0	0	1
Nemegt	13	1	50	23	30	1	4	2	0	1	9
Altan Uul IV	0	4	8	5	4	2	0	1	0	0	3
Altan Uul III	2	1	6	1	5	0	0	0	0	0	2
Altan Uul II	3	2	10	8	11	1	0	3	0	0	7
Guriliin Tsav	2	0	4	3	4	0	0	0	0	0	4
Bugiin Tsav	13	5	22	9	19	3	0	2	0	1	4
Ulaan Khushu	0	0	3	4	6	0	0	1	0	0	1
Tsaagan Khushu	1	0	4	1	5	1	0	0	0	0	1

## 2 Experimental design, materials and methods

Additional details on the methods and materials are available in Funston et al. [1].

### 2.1 Data collection

Map data (Fig. 1, Supplementary File 1) were collected from 358 fossil sites marked by handheld GPS by the first and third authors. Each of these sites comprises a single skeleton identifiable to genus, most of which are articulated, but some are less complete. These were combined with an extensive literature review, focusing mostly on the results of the multi-year Polish Mongolian Palaeontological Expeditions and Hiyashibara Museum Expeditions [2], [3], [4], [5], [6], [7], [8], [9], [10], [11], [12], [13], [14], [15], [16], [17], [18], [19]. Data from these sources was compiled and incorporated into the mapped dataset. Counts were generated for eleven named localities, each of which is a discrete region of outcrop. These localities are: Altan Uul II, Altan Uul III, Altan Uul IV, Bambuu Khudag, Bugiin Tsav, Guriliin Tsav, Hermiin Tsav, Khulsan, Nemegt, Tsagaan Khushuu, and Ulaan Khushuu.

### 2.2 Taphonomic considerations

Taphonomy was not directly addressed during data collection, because lithological data were not always recorded with map data, nor with all the specimens reported in the literature. The approach of grouping specimens by locality partly alleviates this issue, because it allowed us to sample from a wider range of taphonomic modes, and provided a time-averaged assemblage that is representative of palaeocommunity structure [20], [21]. Future work may find success in correcting species counts using other lines of evidence (eggshell, footprints, or microsites).

### 2.3 Taxonomic considerations

There are four theropod, one hadrosaur, and three protoceratopsian taxa that are each represented by a single specimen, and are interpreted variably. *Bagaraatan ostromi* [22] has been considered a troodontid [23], tyrannosauroid [24], or indeterminate coelurosaur [25]. *Borogovia gracilicrus* [26] is a troodontid that may be synonymous with *Saurornithoides mongoliensis* [27]. *Hulsanpes perlei* [28] is probably a deinonychosaur [27] but is not distinctive enough to identify. *Tochisaurus nemegtensis* [29] is a troodontid metatarsus that does not overlap significantly with the material of *Borogovia gracilicrus* or *Zanabazar junior* [30]. It is possible that all three of these troodontid taxa are synonymous, a possibility considered by both Osmólska [26] and Norell et al. [30].The hadrosaur *Barsboldia sicinskii* is known from a partial pelvis and tail. It has been interpreted as a valid taxon [31], a lambeosaurine [32], or as a nomen dubium [33]. Four protoceratopsians have been named from the Baruungoyot deposits in the Nemegt Basin. *Bagaceratops* and ‘*Platyceratops’* are known from Hermiin Tsav, and ‘*Breviceratops’* and ‘*Lamaceratops’* are from Khulsan. Makovicky and Norell [34] suggest that ontogeny can explain all the variation between these four taxa, and synonymize the other three with *Bagaceratops*. Determining the true affinities of these taxa is beyond the scope of this study, so they are not included in the species-level analysis, to avoid numerous single-occurrence taxa. These taxa were, however, included in the superfamily-level data. For these analyses, *Ba. ostromi* was included as a tyrannosauroid. The status of *Opisthocoelicaudia skarzynskii* is questionable, as indicated by Currie et al. [35]. It is likely that most, if not all, material assigned to *Opisthocoelicaudia* will eventually be subsumed into *N. mongoliensis*. Accordingly, all sauropod material from the Nemegt Formation, most of which cannot be identified to species, is treated as *N. mongoliensis*. Indeterminate material that was identifiable to superfamily but not species was also included in the data set. Where only one species of that superfamily is considered valid (i.e. dromaeosaurids and therizinosaurs), indeterminate material was lumped with that species. Where more than one species is considered valid (i.e. ankylosaurs and oviraptorids), indeterminate material was included together as an indeterminate operational taxonomic unit (i.e. Ankylosauria indet., Oviraptoridae indet.).

### 2.4 Statistical methods

Data were not filtered or modified in any way before analysis. Statistical methods used to analyze community composition and taxon distribution in Funston et al. [1] are described therein, in the main text and the Supplementary information.

## Appendix A Supplementary material

The following KMZ files contain the Google maps of the most important areas described in this article.

Map KMZ file containing the Google map.
