# Supplementary material for: A dinosaur community composition dataset for the Late Cretaceous Nemegt Basin of Mongolia
Source: Data Brief. 2017 Dec 6;16:660–6. doi: 10.1016/j.dib.2017.11.086 (PMC5847492; doi:10.1016/j.dib.2017.11.086)
Supplement: Supplementary file 1 — Transparency document [file mmc1.docx]

*Conflict of interest declaration*

**Title: *Dinosaur community composition data for the Late Cretaceous Nemegt Basin of Mongolia***

**Authors: Funston, G. F.^1^, Mendonca, S. E.^2^, Currie, P. J.^1^, Barsbold, R.^3^**

We declare no conflicts of interest, either real or perceived, with the work presented. All appropriate funding agencies have been acknowledged.

Sincerely,

Gregory Funston
